# Contribution of Corticotropin-Releasing Factor Receptor 1 (CRF1) to Serotonin Receptor 5-HT_2C_R Function in Amygdala Neurons in a Neuropathic Pain Model

**DOI:** 10.3390/ijms20184380

**Published:** 2019-09-06

**Authors:** Guangchen Ji, Volker Neugebauer

**Affiliations:** 1 Department of Pharmacology and Neuroscience, Texas Tech University Health Sciences Center, Lubbock, TX 79424, USA; 2 Center of Excellence for Translational Neuroscience and Therapeutics, Texas Tech University Health Sciences Center, Lubbock, TX 79424, USA; 3 Garrison Institute on Aging, Texas Tech University Health Sciences Center, Lubbock, TX 79424, USA

**Keywords:** serotonin, CRF, amygdala neurons, neuropathic pain, short hairpin RNA (shRNA), hyperactivity

## Abstract

The amygdala plays a key role in emotional-affective aspects of pain and in pain modulation. The central nucleus (CeA) serves major amygdala output functions related to emotional-affective behaviors and pain modulation. Our previous studies implicated the corticotropin-releasing factor (CRF) system in amygdala plasticity and pain behaviors in an arthritis model. We also showed that serotonin (5-HT) receptor subtype 5-HT_2C_R in the basolateral amygdala (BLA) contributes to increased CeA output and neuropathic pain-like behaviors. Here, we tested the novel hypothesis that 5-HT_2C_R in the BLA drives CRF1 receptor activation to increase CeA neuronal activity in neuropathic pain. Extracellular single-unit recordings of CeA neurons in anesthetized adult male rats detected increased activity in neuropathic rats (spinal nerve ligation model) compared to sham controls. Increased CeA activity was blocked by local knockdown or pharmacological blockade of 5-HT_2C_R in the BLA, using stereotaxic administration of 5-HT_2C_R short hairpin RNA (shRNA) viral vector or a 5-HT_2C_R antagonist (SB242084), respectively. Stereotaxic administration of a CRF1 receptor antagonist (NBI27914) into the BLA also decreased CeA activity in neuropathic rats and blocked the facilitatory effects of a 5-HT_2C_R agonist (WAY161503) administered stereotaxically into the BLA. Conversely, local (BLA) knockdown of 5-HT_2C_R eliminated the inhibitory effect of NBI27914 and the facilitatory effect of WAY161503 in neuropathic rats. The data suggest that 5-HT_2C_R activation in the BLA contributes to neuropathic pain-related amygdala (CeA) activity by engaging CRF1 receptor signaling.

## 1. Introduction

Serotonin (5-HT) plays an important role in pain modulation and can have facilitatory and inhibitory effects depending on the site of action in the nervous system, cell type, and receptor subtype and affinity [1,2,3,4]. The 14 5-HT receptor subtypes are classified into seven groups based on their structural and functional characteristics [5,6,7]. Selective serotonin reuptake inhibitors (SSRIs) are commonly used in the treatment of major depression and related disorders, and they can relieve neuropathic pain but have limited efficacy and adverse side effects [8,9,10,11,12]. 

The Gq-coupled 5-HT_2C_ receptor (5-HT_2C_R) has been implicated in adverse (anxiogenic) effects of SSRIs [13] and their inconsistent efficacy in neuropathic pain [14]. Preclinical and clinical studies suggest that pharmacological activation of 5-HT_2C_R has anxiogenic effects, while 5-HT_2C_R antagonists are anxiolytic (see references in [15]). Several lines of evidence point to the amygdala, a limbic system structure that plays a key role in emotions and affective disorders [16], as an important site of 5-HT_2C_R-mediated anxiogenic effects. In the amygdala, 5-HT_2C_R messenger ribonucleic acid (mRNA) and protein can be found, particularly in its basolateral nucleus (BLA) [17,18]. The BLA receives serotonergic input from the dorsal raphe nucleus [19,20], and 5-HT release into the BLA, but not central nucleus (CeA), is increased in aversive states [19,21,22,23]. Overexpression of 5-HT_2C_R is found in glutamatergic neurons in the amygdala and other brain areas in calmodulin-dependent protein kinase IIα-2C receptor (CaMKIIα–2CR) transgenic mice [24], and increased 5-HT_2C_R expression in the BLA using recombinant adenovirus-containing 5-HT_2C_R sense sequence [25] resulted in increased anxiety-like behaviors in the elevated plus maze (EPM) and open field test (OFT), which was independent of hypothalamic–pituitary–adrenal (HPA) axis activity [25]. Conversely, 5-HT_2C_R knockout mice showed decreased anxiety-like behaviors and decreased neurochemical activation (c-Fos immunoreactivity) in the CeA [26]. Pharmacologic activation of 5-HT_2C_R in the BLA, but not CeA, had anxiogenic effects in the elevated T-maze, light-dark test and OFT [15,27], and induced ultrasonic vocalizations [27]. A 5-HT_2C_R antagonist (SB242084) in the BLA had anxiolytic effects and blocked anxiogenic effects of local 5-HT or systemic SSRIs in the elevated T-maze [28]. A 5-HT_2A/2C_R antagonist (ritanserin) prevented anxiogenic effects of a systemic 5-HT_2C_R agonist on the EPM [29]. 

The amygdala also plays an important role in emotional-affective aspects of pain and pain modulation [30,31,32]. Synaptic plasticity in the amygdala circuitry composed of CeA neurons and their inputs from the spino-parabrachio-amygdaloid pathway and from the BLA network, has been mechanistically linked to pain behaviors in models of acute [33,34,35,36,37,38,39,40,41,42] and chronic pain [43,44,45,46]. Pain-related synaptic plasticity results in increased background and evoked activity of CeA neurons [38,45,47,48,49,50,51,52,53,54,55] through a mechanism that involves activation of corticotropin-releasing factor 1 (CRF1) receptors in the CeA and BLA [36,48,56]. 

Recent evidence suggests that 5-HT in the amygdala contributes to pain-related neuronal activity changes and behaviors, and these effects are mediated through the 5-HT_2C_ receptor. 5-HT_2C_R has been linked to the neurochemical activation of CeA neurons in an anxiety model (OFT) mentioned previously [26], and 5-HT_2C_R can facilitate synaptic plasticity in BLA pyramidal neurons [57]. 5-HT can also decrease BLA output by direct 5-HT_1A_R-mediated inhibition of glutamatergic principal neurons [58], by HT_1B_R-mediated inhibition of their glutamatergic drive [59] or by 5-HT2R-mediated activation of BLA interneurons [19,58,60]. Recent studies from our group found that knockdown of 5-HT_2C_R in the BLA decreased the excitatory drive of CeA neurons and attenuated nociceptive and averse affective behaviors in a neuropathic pain model [45]. A 5-HT2CR antagonist in the BLA, but not CeA, enabled a systemically applied SSRI to inhibit pain behaviors in an arthritis model [61]. 

The present study tested the novel hypothesis that 5-HT_2C_R in the BLA contributes to neuropathic pain-related hyperactivity in the amygdala output region (CeA) through activation of CRF1 receptors. The results suggest that CRF1 receptor activation downstream of 5-HT_2C_R contributes to increased activity of CeA neurons in a neuropathic pain model. 

## 2. Results

**Figure 1:**
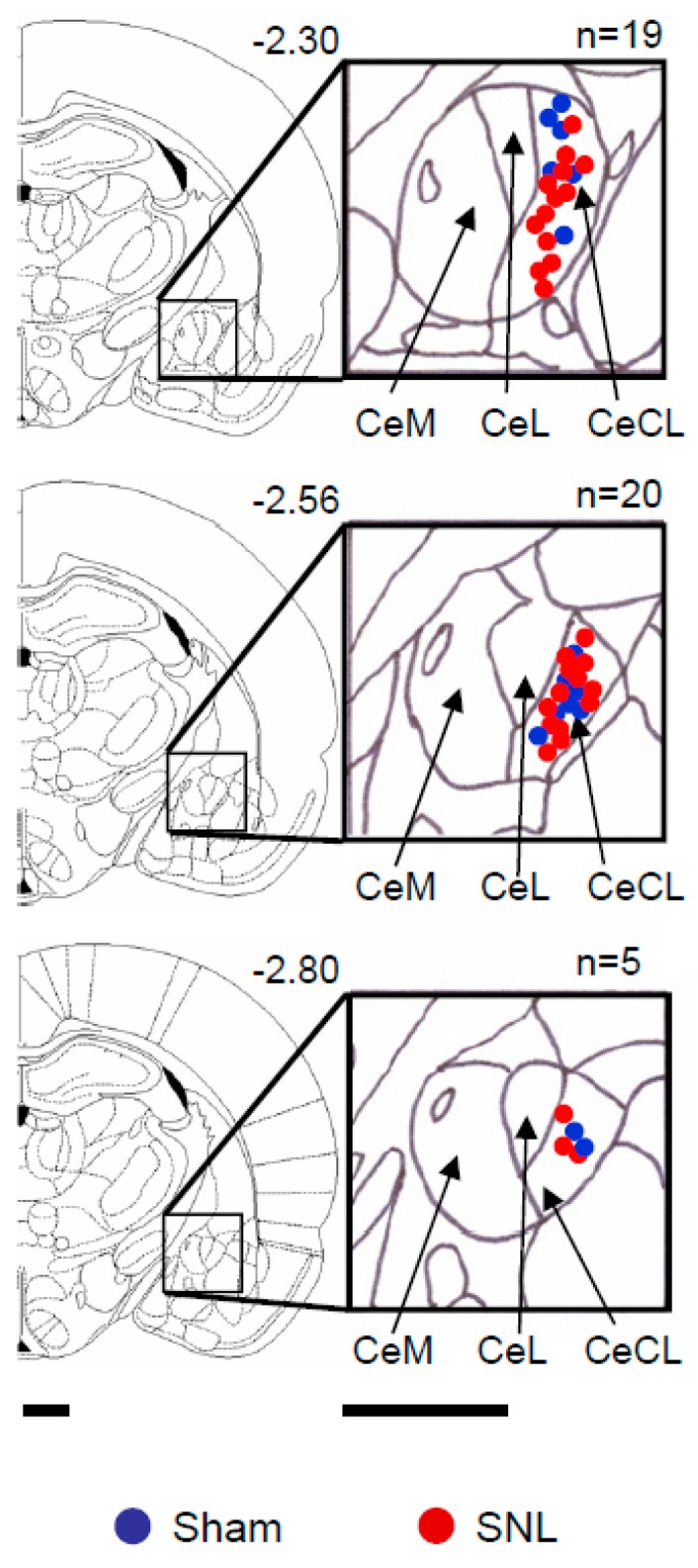
Histologically verified recording sites of 44 neurons in the central nucleus of the amygdala (CeA). The boundaries of the different amygdala nuclei were easily identified under the microscope. Diagrams show the central nucleus and its medial (CeM), lateral (CeL), and latero-capsular (CeLC) subdivisions in coronal sections at different levels posterior to bregma (−2.30 to −2.80). Symbols show the positions of the tips of recording electrodes in the CeA based on electrolytic lesions (see the materials and methods section) in spinal nerve ligation (SNL) (red) and sham (blue) rats. Scale bars, 500 µm.

The experiments described here were designed to analyze the interaction between serotonin receptor 5-HT_2C_R in the basolateral amygdala (BLA) and corticotropin-releasing factor receptor CRF1 signaling downstream of 5-HT_2C_R and their contribution to the increased activity of the central amygdala (CeA) neurons in a neuropathic pain model. Single-unit recordings of CeA neurons in sham control rats and in neuropathic rats (spinal nerve ligation model, SNL) were used. Extracellular single-unit recordings were made from 44 CeA neurons (*n* = 15 neurons in 9 sham rats; *n* = 29 neurons in 11 SNL rats) in anesthetized (isoflurane) adult male rats (Figure 1) as described in the materials and methods section. Neurons were selected that had a receptive field in the left hindpaw (side of sham or SNL surgery), and responded more strongly to brief noxious than innocuous test stimuli; these are so-called “multireceptive” (MR) neurons according to our classification of amygdala neurons [31,45,48,62]. The general experimental protocol was as follows: Induction of pain model (spinal nerve ligation) or sham surgery (Day 0), 5-HT_2C_R short hairpin RNA (shRNA)-enhanced green fluorescence protein (eGFP) for knockdown) or shRNA-eGFP (control) adeno-associated viral vector (AAV) vectors injections (Day 14), and electrophysiological experiments with or without drug applications (Day 28).

### 2.1. 5-HT_2C_R Knockdown in the BLA Inhibits Activity of CeA Neurons in Neuropathic Rats

**Figure 2:**
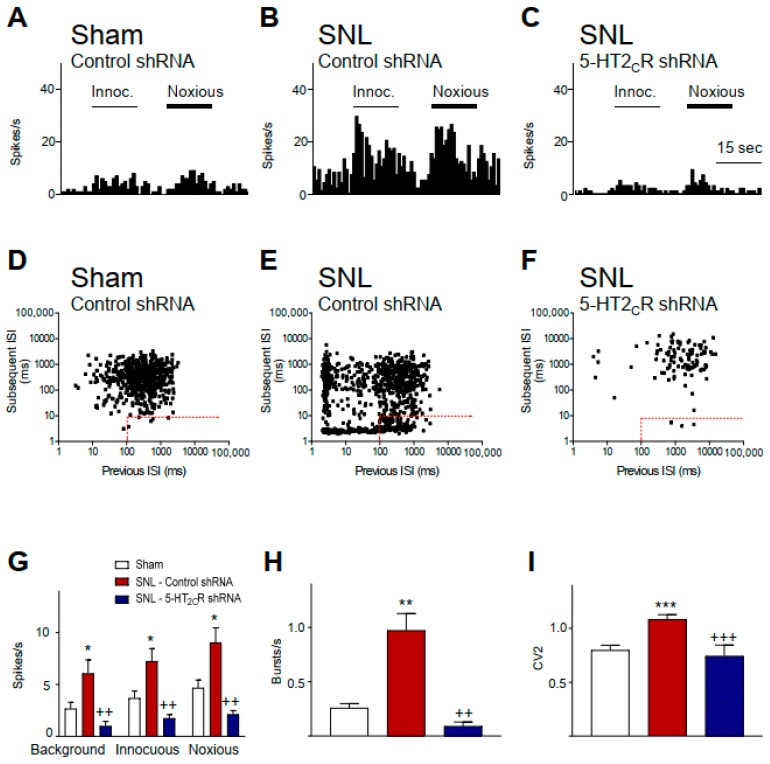
5-HT_2C_R knockdown in the basolateral amygdala (BLA) inhibits CeA neuronal activity in a neuropathic pain model. (**A**–**C**) Examples of individual CeA neurons (three different neurons). Peristimulus time histograms (PSTHs) show the number of action potentials (spikes) per second. Innocuous and noxious stimuli (compression of the hindpaw for 15 s) are indicated by horizontal lines. (**D**–**F**) Joint interspike interval (ISI) plots (previous ISI against the subsequent ISI) detected burst activity, indicated by the rectangular insets (dots within the dashed red lines represent the first spike in a burst). Examples of individual CeA neurons (three different neurons). (**A**,**D**) CeA neuron recorded in a sham rat injected with control vector into the BLA. (**B**,**E**) CeA neuron in an SNL rat with control vector injected into the BLA. (**C**,**F**) CeA neuron in an SNL rat with 5-HT_2C_R knockdown in BLA. (**G**–**I**) Bar histograms showing mean ± SE for the sample of neurons. (**G**) Background activity and net evoked responses to innocuous and noxious stimuli increased significantly in (control vector treated) SNL rats (*n* = 10 neurons) compared to (control vector treated) shams (*n* = 18 neurons). * *p* < 0.05, ANOVA with Bonferroni post hoc tests. 5-HT_2C_R short hairpin RNA (shRNA) vector injected into the BLA (*n* = 6 neurons) decreased background and evoked activity in SNL rats significantly compared to control vector-treated SNL rats. ^++^ *p* < 0.01, ANOVA with Bonferroni post hoc tests. (**H**) Burst activity of CeA neurons was significantly higher in SNL rats (*n* = 10 neurons) compared with shams (*n* = 14 neurons). ** *p* < 0.01, ANOVA with Bonferroni post hoc tests. 5-HT_2C_R knockdown in the BLA decreased burst frequency in SNL rats significantly (*n* = 10 neurons) compared to control vector treated SNL rats (*n* = 18 neurons). ^++^ *p* < 0.01, ANOVA with Bonferroni post hoc tests. (**I**) Irregular firing (CV2) of CeA neurons was significantly increased in SNL rats (*n* = 10 neurons) compared with shams (*n* = 14 neurons). *** *p* < 0.001, ANOVA with Bonferroni post hoc tests. 5-HT_2C_R knockdown in the BLA decreased CV2 in CeA neurons in SNL rats (*n* = 6 neurons) significantly compared to control vector treated SNL rats (*n* = 14 neurons). ^+++^ *p* < 0.001, ANOVA with Bonferroni post hoc tests. (**G**–**I**) Legend for bar histograms in G also applies to H and I.

For local (BLA) knockdown of 5-HT_2C_R, recombinant AAV2 vectors expressing a short hairpin RNA (shRNA) directed at the 5-HT_2C_R or a control hairpin were used [45,63,64]. Either 5-HT_2C_R or a control shRNA-eGFP AAV2 vector was injected stereotaxically into the BLA two weeks after neuropathic or sham surgery, as described in the materials and methods section. Electrophysiology studies were done two weeks after viral vector injection. Compared to CeA neurons in sham controls treated with a control vector (*n* = 18 neurons), CeA neurons in SNL rats treated with control vector (*n* = 10 neurons) showed significantly (*p* < 0.05, ANOVA with Bonferroni post hoc tests) increased background activity and responses to innocuous and noxious stimuli (mechanical compression of the hindpaw with a calibrated forceps, see the materials and methods section; Figure 2G). Individual examples are shown in Figure 2A–C. There was also a significant increase in burst-like activity (Figure 2H; *p* < 0.01) and irregular firing (Figure 2I; *p* < 0.001, ANOVA with Bonferroni post hoc tests) of CeA neurons in SNL rats (*n* = 10 neurons) compared to sham controls (*n* = 14 neurons). Individual examples are shown in Figure 2D–F. Details of the analysis of neuronal activity are described in the materials and methods section. CeA neurons in SNL rats with local 5-HT_2C_R knockdown in the BLA (*n* = 6 neurons) showed significantly lower background and evoked activity (Figure 2G; *p* < 0.01), less burst-like activity (Figure 2H; *p* < 0.01), and less irregular firing (Figure 2I; *p* < 0.001, ANOVA with Bonferroni post hoc tests) compared to CeA neurons in control vector treated SNL rats. Our previous study showed that 5-HT_2C_R knockdown had no effect in sham controls [45]. The results of the present study validate the neuropathic pain-related neuronal changes and the effectiveness of 5-HT_2C_R knockdown observed in our previous study [45], allowing us to use the knockdown strategy to link 5-HT_2C_R and CRF1 receptor function and to confirm the selectivity of pharmacological agents tested here. 

### 2.2. Inhibitory Effects of Antagonists for 5-HT_2C_R and CRF1 Receptor on CeA Neurons in Neuropathic Rats

**Figure 3:**
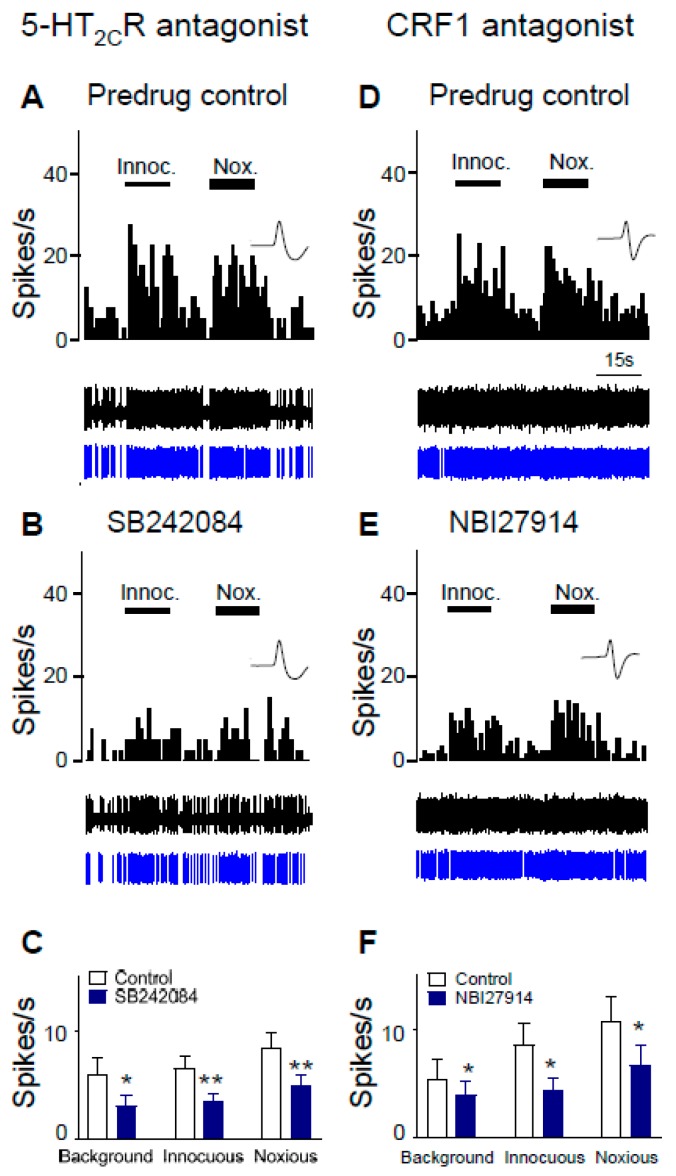
Inhibitory effects of antagonists for 5-HT_2C_R (SB242084) and corticotropin-releasing factor 1 (CRF1) receptor (NBI27914) on CeA neurons in a neuropathic pain model. CeA neurons were recorded in SNL rats 2 weeks after control shRNA was injected into the BLA, i.e., 4 weeks post SNL surgery. Background activity and evoked responses to innocuous and noxious stimulation of the left hindpaw are shown. (**A**,**B**) SB242084 administered into BLA (100 µM concentration in microdialysis fiber; 15 min; (**B**)) decreased background and evoked responses compared to predrug control (ACSF; (**A**)) in an individual CeA neuron. Top traces, peristimulus time histograms (PSTHs) showing the number of action potentials (spikes) per second. Middle traces, original oscilloscope traces. Bottom traces, filtered spikes matched to a preset template that were counted to generate PSTH values. (**C**) Summary of the significant inhibitory effects, SB242084 effects (*n* = 8 neurons). *, ** *p* < 0.05, 0.01, paired *t*-test compared to predrug control (ACSF), respectively. (**D**,**E**) NBI27914 (100 µM concentration in microdialysis fiber; 15 min; (**E**)) decreased background and evoked responses compared to predrug control (ACSF; (**D**)). Same display as in (**A**,**B**). (**F**) Summary of the significant effects of NBI2791 (*n* = 5 neurons). * *p* < 0.05, paired *t*-test compared to predrug control (ACSF).

**Figure 4:**
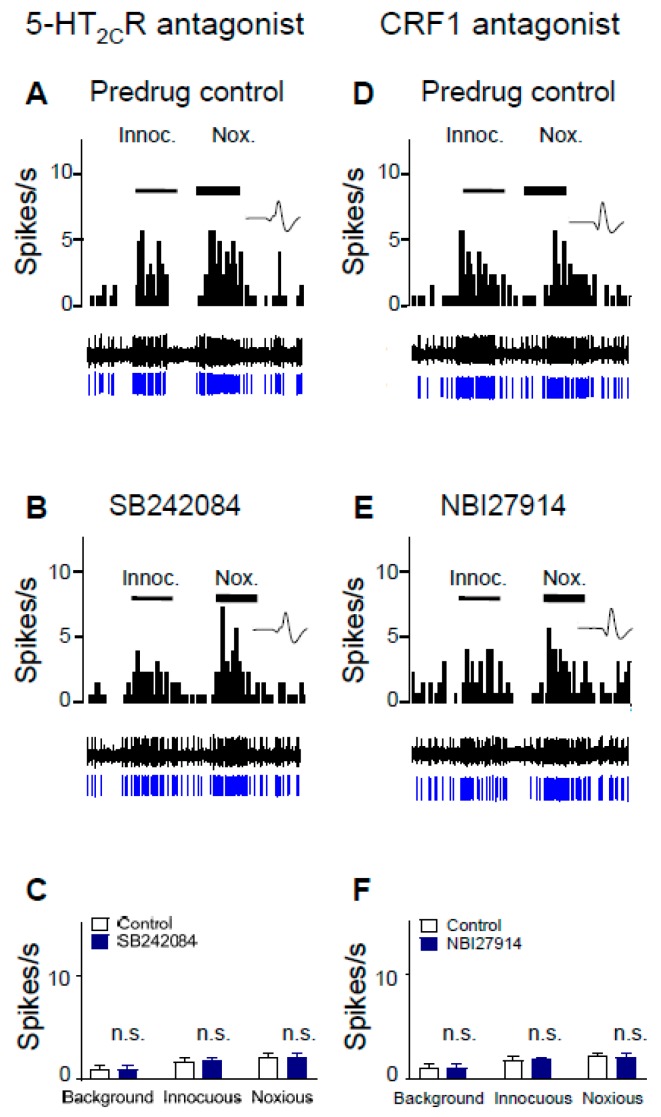
5-HT_2C_R knockdown eliminated the inhibitory effect of antagonists for 5-HT_2C_R (SB242084) and CRF1 receptors (NBI27914) on CeA neurons in a neuropathic pain model. CeA neurons were recorded in SNL rats 2 weeks after 5-HT_2C_R shRNA was injected into the BLA, i.e., 4 weeks post SNL surgery. Background activity and evoked responses to innocuous (100 g/6 mm^2^) and noxious (500 g/6 mm^2^) stimulation of the left hindpaw are shown. (**A**,**B**) SB242084 (100 µM concentration in microdialysis fiber; 15 min; (**B**)) had no effect in an individual CeA neuron compared to predrug control (ACSF; (**A**)). Same display as in Figure 3. (**C**) Summary of lack of significant effects of SB242084 on CeA neurons (*n* = 6 neurons; *p* > 0.05, paired *t*-test compared to predrug). (**D**,**E**) NBI27914 administered into BLA (100 µM concentration in microdialysis fiber; 15 min; (**E**)) had no effect in an individual CeA neuron compared to predrug control (ACSF, (**D**)). (**F**) Summary of the lack of significant effects of NBI27914 on CeA neurons (*n* = 6 neurons; n.s., not significant, *p* > 0.05, paired *t*-test compared to predrug).

The effects of selective antagonists for 5-HT_2C_R (SB242084) and the CRF1 receptor (NBI27914) were tested in neuropathic rats 2 weeks after control shRNA was injected into the BLA (Figure 3). Control shRNA was used to allow the comparison with 5-HT_2C_R knockdown effects shown in Figure 4. Background activity and evoked responses of CeA neurons to innocuous (100 g/6 mm^2^) and noxious (500 g/6 mm^2^) stimulation of the left hindpaw were recorded before (predrug control) and after drug administration. Administration of SB242084 (100 µM, concentration in microdialysis fiber; 15 min) into BLA in SNL rats decreased background activity and evoked responses of CeA neurons significantly (*n* = 8 neurons, *p* < 0.01–0.05, compared to predrug, paired *t*-test; Figure 3C). The activity of an individual CeA neuron was shown before (control, ACSF; Figure 3A) and during antagonist administration (Figure 3B). Administration of NBI27914 (100 µM, concentration in microdialysis fiber; 15 min) into the BLA also decreased background activity and evoked responses significantly (*n* = 5 neurons, *p* < 0.05, compared to predrug, paired *t*-test; Figure 3F). An individual example is shown before (Figure 3D) and during (Figure 3E) antagonist administration. 

### 2.3. 5-HT_2C_R Knockdown Eliminates the Inhibitory Effects of Antagonists for 5-HT_2C_R and CRF1 Receptor on CeA Neurons in Neuropathic Rats

CeA neurons were recorded in neuropathic rats 2 weeks after 5-HT_2C_R shRNA was injected into the BLA to knock down 5-HT_2C_R, i.e., 4 weeks post SNL surgery. In contrast to SNL rats without 5-HT_2C_R knockdown (see Figure 3), administration of SB242084 (100 µM, concentration in microdialysis fiber; 15 min) into the BLA of SNL rats with 5-HT_2C_R knockdown had no significant effect on background activity and on evoked responses of CeA neurons (*n* = 6 neurons, *p* > 0.05, compared to predrug, paired *t*-test; Figure 4C). Activity of an individual CeA neuron before (control, ACSF) and during antagonist administration is shown in Figure 4A,B. Administration of a CRF1 receptor antagonist (NBI27914, 100 µM, concentration in microdialysis fiber; 15 min) into the BLA also had no effect on background and evoked activity of CeA neurons recorded in SNL rats with 5-HT_2C_R knockdown (*n* = 6 neurons, Figure 4F). Figure 4D,E shows an individual example before (control, ACSF) and during antagonist administration. The data suggest that 5-HT_2C_R knockdown in BLA eliminated the inhibitory effects of antagonists for 5-HT_2C_ and CRF1 receptors on CeA neurons in neuropathic pain.

### 2.4. Excitatory Effects of a 5-HT_2C_R Agonist (WAY161503) Are Blocked by a CRF1 Receptor Antagonist (NBI27914) in Neuropathic Rats

**Figure 5:**
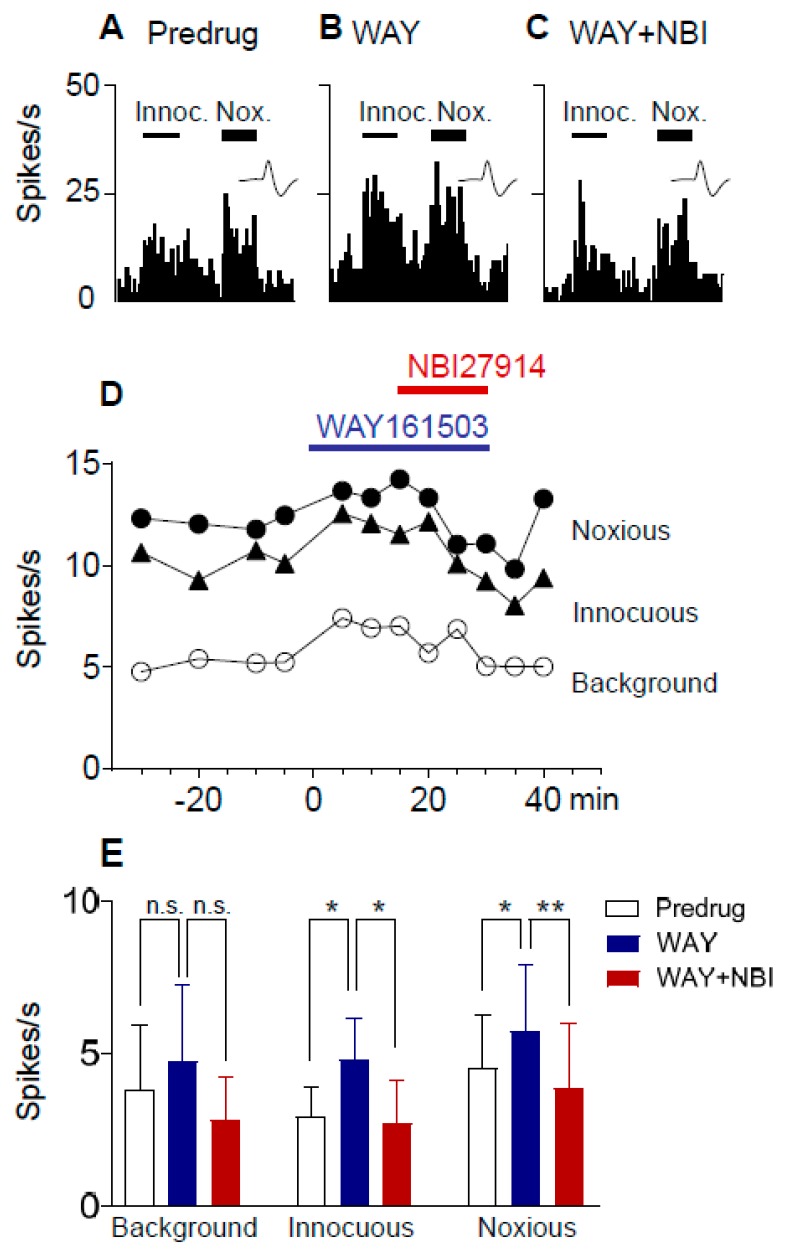
Excitatory effects of a 5-HT_2C_R agonist (WAY161503) were blocked by a CRF1 receptor antagonist (NBI27914) in a neuropathic pain model. CeA neurons were recorded in SNL rats 4 weeks after SNL surgery. Background activity and evoked responses to innocuous (100 g/6 mm^2^) and noxious (500 g/6 mm^2^) stimulation of the left hindpaw are shown. (**A**–**C**) Peristimulus time histograms show action potentials (spikes) per second in one individual CeA neuron before (**A**) and during application of WAY161503 alone (100 µM concentration in microdialysis fiber; 15 min; (**B**)) and during coapplication of WAY161503 with NBI27914 (100 µM concentration in microdialysis fiber; 15 min; (**C**)) into BLA. (**D**) Time course data showing the increase in activity by WAY161503 (100 µM concentration in microdialysis fiber; 15 min) and reversal by NBI27914 (100 µM concentration in microdialysis fiber; 15 min) administered into the BLA. (**E**) Summary of drug effects in SNL rats (*n* = 5 neurons). n.s., not significant; *, ** *p* < 0.05, 0.01 Bonferroni post hoc tests, respectively (repeated measures ANOVA, see results section).

CeA neurons were recorded in neuropathic rats 4 weeks after SNL surgery. Administration of a 5-HT_2C_R agonist (WAY161503; 100 µM, concentration in microdialysis fiber; 15 min) into the BLA increased responses of CeA neurons to innocuous (100 g/6 mm^2^) and noxious (500 g/6 mm^2^) stimulation of the left hindpaw (Figure 5). A selective CRF1 receptor antagonist (NBI27914; 100 µM, concentration in microdialysis fiber) reversed the effect of WAY161503. An individual neuron is shown in Figure 5A–D. Data are summarized in Figure 5E. The facilitatory effects of the 5-HT_2C_R agonist on the responses to innocuous and noxious stimuli, but not background activity, were significant, and the CRF1 receptor antagonist inhibited the facilitation significantly (*n* = 5 neurons; background activity, *p* > 0.05, F_(2,8)_ = 2.686; innocuous, *p* < 0.01, F_(2,8)_ = 9.818; noxious, *p* < 0.01, F_(2,8)_ = 9.914; repeated measures ANOVA). In contrast, WAY161503 had no significant effect in sham rats (*p* > 0.05, paired *t*-test; *n* = 5 neurons; background activity, 1.8 ± 0.69 Hz, predrug; 1.9 ± 0.69 Hz, agonist; responses to innocuous stimuli, 2.1 ± 0.67 Hz, predrug; 2.4 ± 0.93 Hz, agonist; and noxious stimuli, 3.5 ± 1.4 Hz, predrug; 3.3 ± 1.4 Hz, agonist).

### 2.5. Excitatory Effects of a 5-HT_2C_R Agonist (WAY161503) Are Prevented by a CRF1 Receptor Antagonist (NBI27914) in Neuropathic Rats

**Figure 6:**
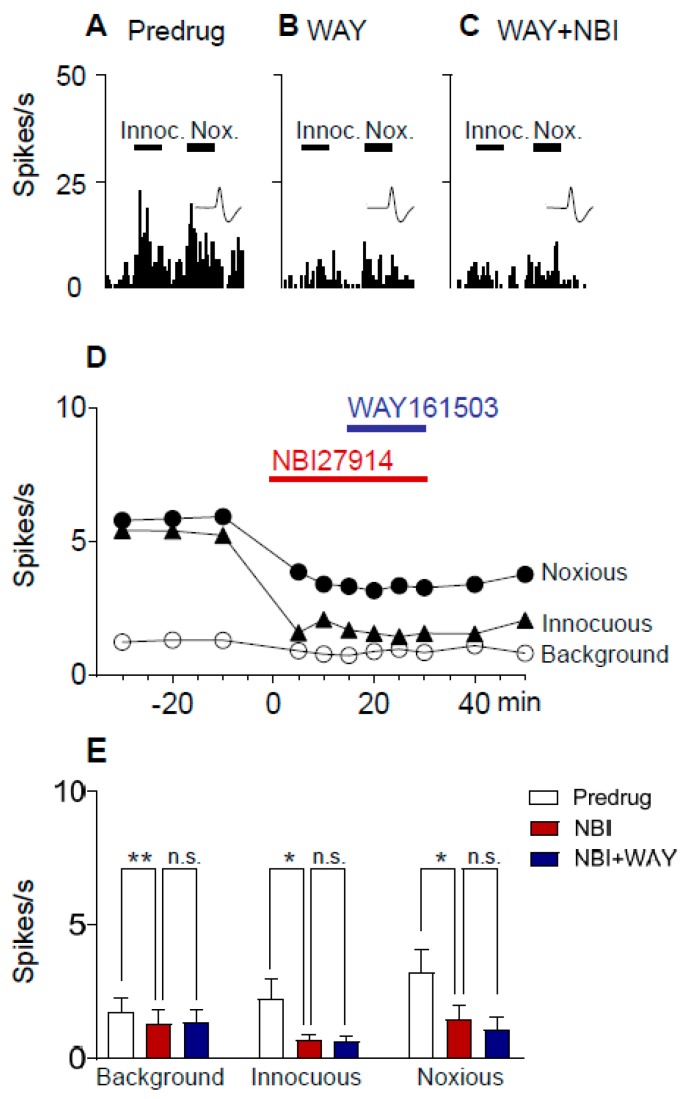
A CRF1 receptor antagonist (NBI27914) prevented the effects of a 5-HT_2C_R agonist (WAY161503) in neuropathic pain. CeA neurons were recorded in SNL rats 4 weeks after SNL surgery. Background activity and evoked responses to innocuous (100 g/6 mm^2^) and noxious (500 g/6 mm^2^) stimulation of the left hindpaw are shown. (**A**–**C**) Peristimulus time histograms show action potentials (spikes) per second in one individual CeA neuron before (ACSF; (**A**)) and during application of NBI27914 (100 µM concentration in microdialysis fiber; 15 min; (**B**)) and during coapplication of NBI27914 with WAY161503 (100 µM concentration in microdialysis fiber; 15 min; (**C**)) into BLA. (**D**) Time course data show the inhibitory effect of NBI27914 (100 µM concentration in microdialysis fiber) and lack of excitatory effect of WAY161503 (100 µM concentration in microdialysis fiber; 15 min) administered into the BLA. (**E**) Summary of drug effects (*n* = 6 neurons). Inhibitory effects of NBI27914 were significant, and continued during co-administration of WAY161503; and WAY161503 had no significant effect. *, ** *p* < 0.05, 0.01, Bonferroni post hoc tests, respectively (repeated measures ANOVA, see results section).

Next, we determined if pre-treatment with a CRF1 receptor antagonist would prevent the 5-HT_2C_R agonist effect. CeA neurons were recorded in SNL rats 4 weeks after SNL surgery. Administration of a selective CRF1 receptor antagonist (NBI27914; 100 µM, concentration in microdialysis fiber) into the BLA decreased background and evoked activity of CeA neurons (Figure 6). In the presence of the CRF1 receptor antagonist, a 5-HT_2C_R agonist (WAY161503; 100 µM concentration in microdialysis fiber; 15 min) had no effect on background activity and evoked responses of CeA neurons. An individual neuron is shown in Figure 6A–D. Data are summarized in Figure 6E. The inhibitory effects of NBI27914 on background activity and responses to innocuous stimuli were significant, and the inhibitory effects persisted during co-administration of WAY161503, suggesting that the CRF1 receptor antagonist prevented the effects of the 5-HT_2C_R agonist (*n* = 6 neurons; background activity, *p* < 0.01, F_(2,10)_ = 14.54; innocuous, *p* < 0.05, F_(2,10)_ = 6.073; noxious, *p* < 0.01, F_(2,10)_ = 10.31; ANOVA).

### 2.6. Excitatory Effects of a 5-HT_2C_R Agonist (WAY161503) Are Eliminated by 5-HT_2C_R Knockdown in Neuropathic Rats

**Figure 7:**
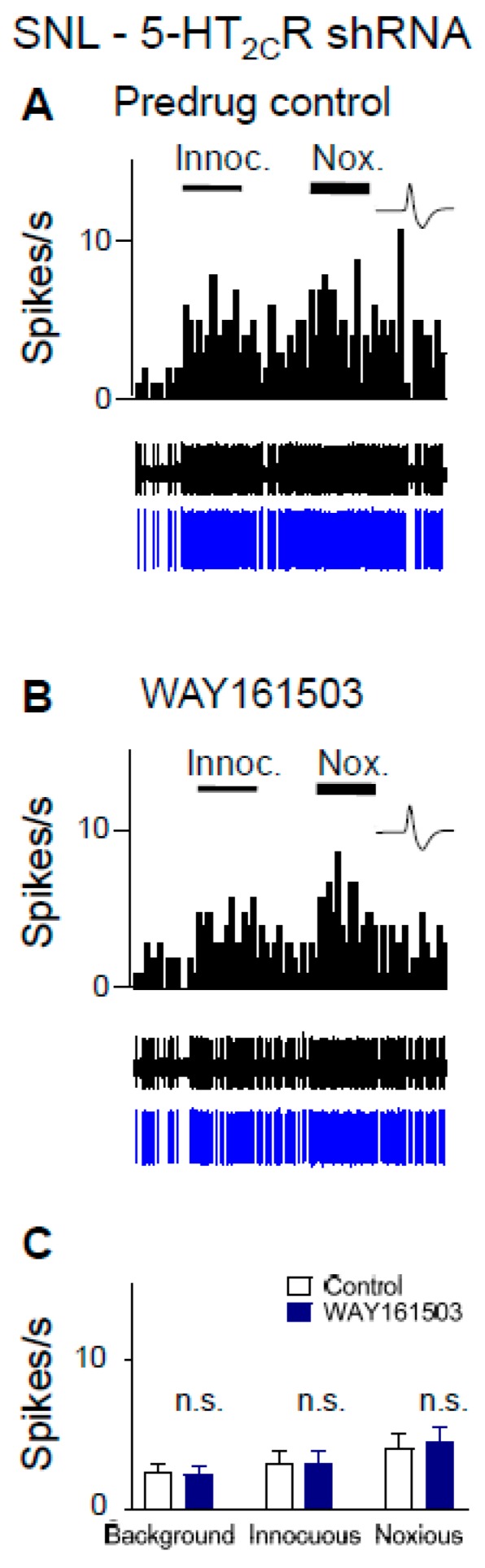
Excitatory effects of a 5-HT_2C_R agonist (WAY161503) were eliminated by 5-HT_2C_R knockdown in neuropathic rats. CeA neurons were recorded in SNL rats 2 weeks after 5-HT_2C_R shRNA was injected into the BLA, i.e., 4 weeks post SNL surgery. Background activity and evoked responses to innocuous (100 g/6 mm^2^) and noxious (500 g/6 mm^2^) stimulation of the left hindpaw are shown. (**A**,**B**) WAY161503 (100 µM concentration in microdialysis fiber; 15 min; (**B**)) had no effect in an individual CeA neuron compared to predrug control (ACSF; (**A**)). Same display as in Figure 3 and Figure 4. (**C**) Summary of lack of significant facilitatory effects of WAY161503 on CeA neurons (*n* = 6 neurons; *p* > 0.05, paired *t*-test compared to predrug).

CeA neurons were recorded in SNL rats 2 weeks after 5-HT_2C_R shRNA was injected into the BLA to knock down 5-HT_2C_R, i.e., 4 weeks post SNL surgery. In contrast to neuropathic rats without knockdown (Figure 5 and Figure 6), WAY161503 (100 µM concentration in microdialysis fiber; 15 min) had no significant facilitatory effects (*p* > 0.05, paired *t*-test; *n* = 6 neurons) in SNL rats with 5-HT_2C_R knockdown (Figure 7C). Activity of an individual CeA neuron before (control, ACSF) and during agonist administration is shown in Figure 7A,B.

## 3. Discussion

Serotonin 5-HT_2C_R has emerged as an important target for the treatment of neurological and psychiatric disorders, including substance use disorders, schizophrenia, impulsive/compulsive disorders, anxiety, depression, and epilepsy. 5-HT_2C_R can modulate multiple neurotransmitter systems to modulate network functions and behaviors, and effects may depend on the site of action in the nervous system, differential coupling to intracellular signaling pathways, and condition/disease (see reference [65]).

While there is evidence for pro-nociceptive and anxiogenic effects of 5-HT_2C_R (see the introduction section and [66]), its role in pain modulatory centers in the brain is an understudied area of research. Evidence from our recent study suggests that 5-HT_2C_R in the basolateral amygdala (BLA) increases neuronal activity in the amygdala output region (central nucleus, CeA) to drive neuropathic pain-like behaviors [45]. Knockdown of 5-HT_2C_R in the BLA decreased the excitatory drive of CeA neurons more strongly than synaptic inhibition and attenuated nociceptive and averse affective behaviors in a neuropathic pain model, whereas 5-HT_2C_R knockdown in sham controls only decreased synaptic inhibition without behavioral effects [45]. Further supporting an important role of 5-HT_2C_R in amygdala-dependent behaviors, a 5-HT2CR antagonist in the BLA, but not CeA, enabled a systemic SSRI to inhibit pain behaviors in an arthritis model [61]. 

The present study advanced this line of research by testing the novel hypothesis that the contribution of 5-HT_2C_R in the BLA to neuropathic pain-related hyperactivity in the CeA involves activation of CRF1 receptors. Increased CeA activity was blocked by local knockdown of 5-HT_2C_R in the BLA (Figure 2) or by a 5-HT_2C_R antagonist (SB242084) administered into the BLA (Figure 3). Stereotaxic administration of a 5-HT_2C_R agonist (WAY161503) had facilitatory effects in the neuropathic pain model (Figure 5) but not in sham controls (see Section 2.4), which is consistent with the results of our previous brain slice physiology study showing a contribution of 5-HT_2C_R in the BLA to excitatory transmission in the pain model but synaptic inhibition under control conditions [45]. 

Next, we addressed the potential contribution of CRF1 receptors. The amygdala (CeA but not BLA) is a major site of CRF expression, showing the highest content of CRF neurons outside the hypothalamus [67,68,69,70,71]. CRF1 receptors are expressed in the CeA and BLA [72] and play an important role in pain-related amygdala plasticity and amygdala-mediated behaviors in models of arthritic, visceral, and neuropathic pain [36,48,56,73,74,75,76,77]. 

There is evidence to suggest that in the amygdala CRF acts downstream of 5-HT_2C_R [26]. Elimination of 5-HT_2C_R in knockout mice blocked the neurochemical (FOS) activation of CRF CeA neurons in response to an anxiogenic stimulus (open field) [26]. The midbrain dorsal raphe nucleus is a major source of serotonergic input to the amygdala, and 5-HT axons innervate pyramidal cells and interneurons in the BLA, but not CeA [20,78,79,80]). 5-HT_2C_R is expressed postsynaptically to serotonergic axons [17,18]. Pharmacologic activation of 5-HT_2C_R in the BLA, but not CeA, increased measures of anxiety-like behaviors in the elevated plus maze [81], elevated T-maze [15], light-dark transition test [15], and open-field test [82]. These lines of evidence point to the BLA as an important site of 5-HT_2C_R action to modulate CeA function.

In the present study, we recorded from CeA neurons to determine their modulation by 5-HT_2C_R through CRF1 receptors in the BLA. The BLA receives polymodal, including nociceptive, information and provides synaptic excitation as well as feedforward inhibition to CeA neurons [30,31,32,83]. While the CeA is generally considered the output region for major amygdala function [70], including emotional-affective aspects of pain and pain modulation [30,31,32,84], the BLA has also been implicated in top-down pain modulation [85,86], averse affective aspects of pain [83], and cognitive deficits associated with pain [56]. Our recent study showed a 5-HT_2C_R-driven shift towards excitatory influences of the BLA on CeA activity and behaviors in a neuropathic pain model [45]. 

Here, we report that a CRF1 receptor antagonist administered into the BLA blocks or prevents (Figure 5 and Figure 6) the increase of CeA neuronal activity by a 5-HT_2C_R agonist (WAY161503) administered into the BLA in the neuropathic pain model (Figure 5), suggesting that 5-HT_2C_R activation generates endogenous activation of CRF1 receptors, presumably through the activation of CRF neurons in the CeA [67,68,69,70,71] and release of CRF into the CeA and BLA. 5-HT_2C_R knockdown in the BLA eliminated the agonist effects (Figure 7), confirming that they were mediated by 5-HT_2C_R. 5-HT_2C_R knockdown in the BLA of neuropathic rats also blocked the inhibitory effects of a CRF1 receptor antagonist (Figure 4), providing further support of our hypothesis that 5-HT_2C_R activation in the BLA contributes to neuropathic pain-related amygdala (CeA) activity by engaging CRF1 receptor signaling.

As a note of caution, we did not confirm the effects of 5-HT_2C_R knockdown in this study. The viral vector that we used has been validated thoroughly [63,64] and we showed previously that focal (BLA) injection of AAV-expressing 5-HT_2C_R shRNA, but not control shRNA, decreased 5-HT_2C_R mRNA and immunoreactivity [45]. Using the same tools, assays, and protocol, there was no reason to expect different results. Another consideration is drug application by microdialysis. This technique offers several advantages, such as avoidance of volume effect and steady state application, but the exact tissue concentration can only be estimated (see the materials and methods section). We selected concentrations based on our previous studies using antagonists for the CRF1 receptor (NBI27914) [36,56,87,88] and 5-HT_2C_R (SB242084), which was supported by data in the literature (see the materials and methods section). Importantly, local (BLA) knockdown of 5-HT_2C_R eliminated the inhibitory effect of SB242084 (Figure 4) and the facilitatory effects of a 5-HT_2C_R agonist (WAY161503) (Figure 7) in neuropathic rats, which strongly argues for specific actions on 5-HT_2C_R. Finally, we did not perform off-site drug injections in this study. However, we showed previously that 5-HT_2C_R knockdown in the BLA, but not the CeA, had inhibitory neuronal and behavioral effects [45]. Data in the literature also support an action of 5-HT_2C_R in the BLA, but not the CeA (see the introduction and discussion sections). In this study, 5-HT_2C_R knockdown in the BLA blocked the effects of pharmacological agents (Figure 4 and Figure 7). While CRF1 receptors are expressed and functional both in BLA and CeA [36,56], offsite injections of CRF or CRF1 and CRF2 receptor antagonists into the neighboring striatum had no effect [36,87]. These findings suggest that 5-HT_2C_R in the BLA and CRF1 in the amygdala contributed to the observed effects on the CeA neurons, but the site of action of CRF1 in the BLA versus CeA could not be distinguished here.

In summary, the new results of this study link CRF1 receptor activation to 5-HT_2C_R in the BLA in a neuropathic pain model. The 5-HT_2C_R and CRF1 receptor interaction results in increased activity of CeA neurons. Our previous work showed an important role of 5-HT_2C_R in the BLA for CeA neuronal hyperactivity in neuropathic pain. The new data show that 5-HT_2C_R knockdown eliminates the inhibitory effects of a CRF1 receptor antagonist and the facilitatory effects of a 5-HT_2C_R agonist, and that the agonist effects are also blocked or prevented by a CRF1 receptor antagonist. 

## 4. Materials and Methods

### 4.1. Animals

Male Sprague Dawley rats (250–350 g, Harlan Laboratories) were housed in a temperature-controlled room in the animal facility under a 12 h light/dark cycle with unrestricted access to water and food. On the day of the experiment, rats were transferred to the laboratory and allowed to acclimate for at least 1 h. All experimental procedures were approved by the Institutional Animal Care and Use Committee (IACUC) at Texas Tech University Health Sciences Center, TX, USA (Protocol 14006, approved 23 June 2014) and conformed to the guidelines of the International Association for the Study of Pain and of the National Institutes of Health. 

### 4.2. Neuropathic Pain Model

The spinal nerve ligation (SNL) model [89] was used as described in our previous study [45]. Rats were anesthetized with isoflurane (3–4% induction, 2% maintenance) while the L5 spinal nerve was tightly ligated using 6–0 silk, using sterile techniques. In the sham control group, the spinal nerve was exposed but not ligated. The SNL model generates stable neuropathic pain behaviors that last for at least 4–5 weeks [45]. While the experimenter was not blinded to the experimental condition for technical reasons, the analysis involved a blinded experimenter.

### 4.3. Viral Vector for 5-HT_2C_R Knockdown

For local (basolateral amygdala, BLA) knockdown of 5-HT_2C_R, recombinant AAV2 vectors expressing a short hairpin RNA (shRNA) directed at the 5-HT_2C_R or a control hairpin were used [45,63,64]. Vectors were obtained from the Vector Core at the University of North Carolina at Chapel Hill (UNC Vector Core). 5-HT_2C_R or control shRNA-eGFP AAV2 vector (1 µL) was injected stereotaxically into the BLA of rats anesthetized with isoflurane (3–4% induction, 2% maintenance; precision vaporizer, Harvard Apparatus), two weeks after neuropathic or sham surgery. The following coordinates were used: 2.5 mm caudal to bregma, 4.8 mm lateral, 8.0–8.5 mm depth. Two weeks after neuropathic surgery, control shRNA-eGFP AAV vector (1 µL) or 5-HT_2C_R shRNA-eGFP AAV vector (for 5-HT_2C_R knockdown; 1 µL) was injected stereotaxically into the BLA. Rats recovered for 2 weeks before the electrophysiological recordings to allow for stable transgene expression. 5-HT_2C_R knockdown was validated with qPCR, western blot analysis and immunohistochemistry in our previous study [45]. 

### 4.4. Systems Electrophysiology

Extracellular single-unit recordings were made from neurons in the laterocapsular division of the CeA (CeLC) as described previously ([90], for recent references see [45]). Rats were anesthetized with isoflurane (3–4% induction, 2% maintenance). Core body temperature was maintained at 37 °C with a homeothermic blanket system. The animal was mounted in a stereotaxic frame (Kopf Instruments) and a craniotomy was performed at the sutura frontoparietalis level to allow the insertion of the recording electrode and microdialysis probe for drug or vehicle administration. 

Neurons were recorded with glass-insulated carbon filament electrodes (4–6 MΩ) using the following coordinates: 2.3–2.8 mm caudal to bregma, 3.8–4.2 mm lateral to midline, depth 7–8 mm. The recorded signals were amplified, band-pass filtered (300 Hz to 3 kHz), displayed on an analog oscilloscope, and processed by an interface (1401 Plus; CED). Spike2 software (version 4; CED) was used for spike sorting, data storage, and analysis of neuronal activity. Spike size and configuration were monitored continuously. For each neuron, a spike template was created during a 5 min baseline recording period. Only those neurons were included in the study that showed a spike configuration that matched the preset template and could be clearly discriminated from activity in the background throughout the experiment. Neurons were identified by monitoring background activity and responses to search stimuli, i.e., compression of the hindpaw at innocuous (100 g/6 mm^2^) and noxious (500 g/6 mm^2^) intensities with calibrated forceps. Noxious stimuli were used sparingly. Neurons included in this study had a receptive field in the hindpaw (L5 territory) and were activated more strongly by noxious than innocuous mechanical stimuli. One neuron was recorded per animal to allow for verification of the recording with electrolytic lesion at the end of the experiment (see “verification of recording site”) and to avoid confounding effects of drug applications.

### 4.5. Neuronal Activity Analysis

Background activity (in the absence of intentional stimulation) was measured for 5 min and evoked activity was recorded during brief (15 s) innocuous and noxious test stimuli applied to the hindpaw. Net evoked activity was calculated by subtracting background activity (mean value of 1–3 min) preceding the stimulus from the total activity during stimulation. Neuronal activity was expressed as spikes per second. Spike2 software burst analysis script was used to analyze interspike interval (ISI) distribution and burst-like activity for each CeA neuron, as described previously [45]. Burst-like activity was defined as in our previous study [45]—a silent period of at least 100 ms preceded the first spike in a burst, which was followed by a second spike with an ISI of ≤10 ms. The coefficient of variation (CV2) was calculated to detect irregular firing based on the variation in the ISIs. CV2 was calculated as described previously [45] by assessing the SD and mean firing for two adjacent ISIs (CV2 = 2 (ISI2−ISI1)/(ISI2+ISI1)). The CV2 for each pair of adjacent ISIs was plotted against the mean of those two ISIs. Small CV2 values indicate regular firing, whereas large CV2 values (≥1) indicate irregular firing.

### 4.6. Drugs and Drug Application by Microdialysis

A 5-HT_2C_ receptor agonist (WAY161503), a 5-HT_2C_ receptor antagonist (SB242084), and a CRF1 receptor antagonist (NBI27914) were purchased from Tocris Bioscience. Several hours before the start of the electrophysiological recordings, a microdialysis probe (CMA11/Microdialysis; 250 µm membrane diameter; 1 mm membrane length) was lowered vertically into the BLA, ipsilateral to the recording electrode, using the following coordinates: 2.5 mm caudal to bregma, 4.8 mm lateral to midline, 9.0 mm depth. The distance between the microdialysis probe and recording electrode was ~0.5 mm. Before each drug application, artificial cerebrospinal fluid (ACSF) was pumped through the microdialysis fiber for about 1 h at a rate of 5 µL/min to establish equilibrium in the tissue. The microdialysis probe was connected to an infusion pump (Harvard) using PE-50 tubing. The ACSF contained (in mM) 125.0 NaCl, 2.6 KCl, 2.5 NaH2PO4, 1.3 CaCl2, 0.9 MgCl2, 21.0 NaHCO3, and 3.5 glucose oxygenated and equilibrated to pH ± 7.4. Drugs were dissolved in ACSF on the day of the experiment at a concentration of 100 times that was predicted to be needed based on data from our previous studies [36,48,61,91,92] and data in the literature [93,94,95]. Drugs were administered into the BLA at a rate of 5 µL/min. 

### 4.7. Verification of Recording Site

At the end of each experiment, the recording site in the CeA was marked by an electrolytic lesion with DC (250 mA for 3 min) injected through the recording electrode. The brain was removed and submerged in 10% formalin and potassium ferrocyanide. Tissues were stored in 30% sucrose before they were frozen sectioned at 50 µm and stained with hematoxylin and eosin. The boundaries of the different amygdala nuclei were easily identified under the microscope. Lesion/recording sites were verified histologically and plotted on standard diagrams.

### 4.8. Statistical Analysis

All averaged values are given as the mean ± SE. Statistical significance was accepted at the level *p <* 0.05. GraphPad Prism 5.0 software was used for all statistical analyses. Statistical analysis was performed on the raw data.
